# Ecological Aspects of the Vector-Borne Bacterial Disease, Citrus Greening (Huanglongbing): Dispersal and Host Use by Asian Citrus Psyllid, *Diaphorina Citri* Kuwayama

**DOI:** 10.3390/insects10070208

**Published:** 2019-07-16

**Authors:** Lukasz L. Stelinski

**Affiliations:** Department of Entomology and Nematology, Citrus Research and Education Center, IFAS, University of Florida, 700 Experiment Station Road, Lake Alfred, FL 33850, USA; stelinski@ufl.edu; Tel.: +1-863-9568-851

**Keywords:** movement, landscape, vector exclusion, pest management, phytopathogens

## Abstract

Determining the influence of abiotic and biotic factors on pest dispersal behavior is a critical component of integrated pest management. The behavioral and physiological traits of movement of the Asian Citrus Psyllid (ACP), *Diaphorina citri* Kuwayama, has received significant attention. Field and laboratory experiments have explored the physiological capabilities of ACP dispersal, as well as, the abiotic and biotic drivers that initiate movement behavior. Abiotic factors such as temperature, barometric pressure, humidity, landscape, and orchard architecture, as well as, biotic factors including mating status, pathogen infection, and morphotype have been investigated in great detail. The current review focuses on dispersal of ACP with the goal of synthesizing current knowledge to suggest management tactics. Overall, vision serves as the primary modality for host finding in ACP. Current data suggest that ACP populations increase more within uniform landscapes of seedling trees, as compared to mature orchards with randomly interspersed young seedlings. The data also suggest that establishment and conservation of visual and physical barriers might be beneficial to protect orchards from ACP. Management of ACP must take into account large-area cooperation, orchard border surveillance and treatment, removal of non-crop habitat, and an understanding that immigration can occur from distances of several kilometers.

## 1. Introduction

During the past decade and a half, research dedicated to the Asian citrus psyllid (ACP), *Diaphorina citri* Kuwayama (*Candidatus* Liberibacter sp.) huanglongbing (HL) pathosystem has expanded significantly. This is due to the economic toll that huanglongbing has imposed on global citrus production and the efforts required to mitigate it. Research efforts have been diverse and have included all aspects of the disease triangle with a “no stone left unturned approach”. Fundamental understanding of the photosystem has increased significantly and new tools and practices have emerged to help with preventing disease spread to new areas and mitigating the negative impact of the disease where it is endemic. However, to date, there remain no cures and the disease continues to impose a significant economic burden on citrus production, worldwide. Several excellent reviews of recent literature are available that have summarized and synthesized new and old information on this pathosystem (see discussion below). To a large degree, many of these reviews remain current, despite the astonishing pace with which new publications on HLB emerge, and this paper is not intended to replace them. Instead, the purpose of this mini-review is to specifically focus on vector-pathogen-HLB interactions as they relate to vector movement and dispersal behavior. Management of HLB requires a holistic approach and attention to the complete disease triangle. However, a thorough understanding of vector dispersal and movement is critical for development and implementation of management plans that aim to either exclude or treat this disease.

## 2. Host Specificity

### 2.1. Host Range

Previous comprehensive reviews and summaries of ACP biology indicate a broad host range within the rutacious subfamily, Aurantioideae (reviewed in [1]), [2]. Oviposition and development is similar on all commonly grown citrus cultivars and related orange jasmine, *Murraya paniculata* (L.) with apparent differences relating mainly to amount of new leaf growth [3,4]. At least ten genera in addition to citrus are known host plants [5]. More recently, investigations have focused on identification of citrus and citrus-related genotypes that display resistance to colonization or subsequent development by ACP. In apparent exception to the above rule, oviposition, development, and survival of ACP is reported to be significantly lower on ‘Sunki’ mandarin (*Citrus sunki*) [4] and ‘Cleopatra’ mandarin (*Citrus reshni* Hort. ex Tan.) [6], than on other known suitable host plants. In addition, ACP significantly avoid colonizing trifoliate orange, *Poncirus trifoliata* (L.), and completely avoid colonizing the citrus-related species, white sapote (*Casimiroa edulis* Llave et Lex) [7]. Given that trifoliate orange readily hybridizes with *Citrus* spp., it might be a promising candidate for citrus breeding efforts that aim to develop cultivars expressing partial resistance to ACP [7].

### 2.2. Alternative Hosts

ACP predominantly reproduce on plants in the Rutaceae family; reproduction on *Ficus carica* L. (Moraceae) [8], and feeding on hackberry (*Celtis* spp.) and potatoes *Solanum tuberosum* L. [9] has also been confirmed. Most recently, additional secondary hosts of ACP have been identified in Florida, including *Bidens alba* (L.) and *Eupatorium capillifolium* (Lam.), which are common weed species occurring in citrus orchards [10]. Therefore, the breadth of alternative hosts reaches beyond the Rutaceae. Although some of these alternative hosts are not suitable for reproduction, they might serve as hosts for adult feeding, prolonging survival in the absence of reproductive hosts. This strategy might explain long distance movement impacting colonization of citrus orchards [11]. ACP have been captured in locations several kilometers away from known host plants [12]. In the absence of choice, ACP can survive on gallberry *Ilex glabra* (L.) Gray, Darrow’s blueberry *Vaccinium darrowii* Camp, and redbay, *Persea borbonia* (L.) Spreng, for up to 7 days

## 3. Dispersal

### 3.1. Seasonal Dispersal Patterns

Local dispersal of ACP was found to be greatest during the spring and summer months and decreased greatly during the colder months (September–March) [13,14]. During the warmer months, ACP consistently dispersed distances of at least 300 m, within four days, from inner rows of abandoned orchards to inner rows of managed orchards [14]. Hall and Hentz [13] reported similar results showing psyllid movement areas of up to 150 m away from the citrus orchards. Interestingly, Lewis-Rosenblum et al. [14] captured fewer psyllids that were marked in situ, with proteins, in abandoned citrus orchards than in adjacent commercial orchards, over the course of two growing seasons. This suggests that psyllids moved longer distances to find more suitable hosts rather than moving to new sub-optimal hosts within their proximate location.

ACP can disperse from abandoned areas into nearby commercial orchards within only a few days [15]. In addition, Lewis-Rosenblum et al. [14] suggested that ACP disperse less in winter. ACP adults were present in the orchards, as indicated by tap counts, but in-situ-(protein)-marked psyllid captures on traps, declined to zero from October 2009–March 2010. This was congruent with Hall and Hentz [13], who documented only short distance movements (2 m from citrus trees) of ACP, during winter.

### 3.2. Dispersal Range

In-field marking and recapture of wild ACP showed that adults can disperse >2,000 m within 12 days [14]. Long distance travel by *D. citri* occurs in search of required resources, such as unfurled, emerging leaves. ACP movement is primarily affected by availability of new leaves and insecticide spray applications [14]. New leaves are required for ACP reproduction, and thus, it is not surprising that this affects ACP movement. ACP are known to avoid insecticides. Hall and Hentz [13] also noted that abundance of new leaves affects dispersal, with fewer psyllids trapped at locations with decreasing amount of this resource, as measured from the point of release.

ACP move pathogen during dispersal between Las-infected and uninfected trees [16,17]. Therefore, the rate and range of HLB dissemination is directly affected by the dispersal range of ACP [1]. ACP can fly continuously up to 2.4 km in the absence of wind [18]. Long-range dispersal is likely enhanced by wind. Gottwald et al. [19] hypothesized that the dispersal range of ACP due to wind assistance could be from 90–145 km. Sakamaki [20] proposed that ACP could have dispersed up to 470 km, throughout the Okinawan islands, due to a lower jet stream movement. Kobori et al. [21] documented 5–12 m dispersal distances with mark–release–recapture and suggested that ACP move infrequently for the initial few days following colonization of a host plant. However, in Florida, ACP disperse >100 m between citrus orchards [15]. Hall and Hentz [13] documented psyllid dispersal up to 150 m with peak movement occurring during spring. Adult ACP can also move at least 300 m, over fallow ground [14,15].

### 3.3. Biotic Drivers of Dispersal

#### 3.3.1. Visual Cues

ACP are prominently phototatic and aggregate near sources of intense light [22,23]. ACP adults are most attracted by ultraviolet (390 nm), green (525 nm), and yellow (590 nm) light and are most active in the afternoon [24]. This behavior can be exploited practically by employing windbreaks that decrease ACP infestation on border rows of citrus orchards [25] or reflective mulches that disorient host-seeking by ACP in citrus replants [26].

#### 3.3.2. Mechanical Cues

Close-range sexual communication among male and female ACP occurs by substrate-borne vibration [27,28].

#### 3.3.3. Olfactory Cues

ACP are attracted to leaf volatiles of various citrus species, which in some cases have been identified by gas chromatography–mass spectrometry (GC–MS) [29]. Wenninger et al. [30] demonstrated that ACP response to plant odors is affected by mating status, host plant variety, and color. Electroantennogram studies demonstrated antennal responses by adult ACP to degradation products of some common host plant volatiles, even when the parent molecules do not elicit antennal responses [31]. Attractiveness to ACP in the blend reported by Coutinho-Abreu et al. [32] remains to be confirmed under field conditions in Florida. The most consistent attractant known for this psyllid in the field is the color yellow [33] and, thus, visual behavior is likely the principle driver of long-range host attraction.

#### 3.3.4. Overcrowding

Odors from ACP females repel female conspecifics, but not males [34]. Female ACP also detect and avoid leaves that were previously infested by other *D. citri* females [34]. Therefore, a chemical is likely deposited on plants during infestation, which is detected and avoided. This odor could be related to the male-attractant pheromone suggested by Wenninger et al. [35] or could be derived from the cuticular hydrocarbon(s) characteristic of female ACP that attract conspecific males [36]. Repellency between conspecific males has been previously documented in winterform pear psylla [37], but female-female repellency was not tested in this case.

#### 3.3.5. Mate Finding

Male ACP are attracted to females [35]. ACP females are also attracted by volatiles emitted by plants that were damaged by conspecific feeding [34]. Feeding by ACP on citrus induces the release of methyl salicylic acid (MeSA), which attracts ACP at the specific dosage released by psyllid-damaged plants [38]. Therefore, this semiochemical might attract female ACP to damaged plants. The ecological benefit of female ACP attraction to psyllid-damaged plants might be the requirement of multiple matings, to maintain optimal fertility and fecundity [39]. Thus, females consistently require male mating partners [39] and plants that emit damage-induced volatiles might facilitate host detection [40] and mate finding. Density-dependent repellency among female ACP combined with attraction to damage-induced plant volatiles might improve the fitness of offspring.

#### 3.3.6. Host Plant Experience

ACP develop preference for the citrus genotypes on which they developed [41]. This can be observed by differences in oviposition preference, following differential host plant experience [41,42,43]. Overall, adult ACP prefer to settle and lay eggs on the host plant genotypes on which they developed and maternal preference might influence offspring fitness [41].

ACP show a significant amount of behavioral plasticity and learning behavior. Males learn to prefer odor of certain females, following previous experience [42], and similarly females learn to prefer certain male phenotypes that appears to be adaptive [43]. This is consistent with previous studies, which have quantified differences in ACP development and morphological features, depending on the host plant genotype on which they developed [44,45].

#### 3.3.7. Color Morphotype

Differences in the abdominal color of ACP are related to several measurable phenotypic differences. Gray/brown ACP are poorer fliers than green/blue psyllids [18]. While most ACP can fly continuously between 1 to several minutes [46], some blue/green ACP morphs can fly continuously for up to 3 h [18]. Gray/brown psyllids are generally smaller than blue/green morphs [18,47,48]. The effect of pigmentation differences on flight performance of hemipterans insects is poorly understood. Differences between the red and green pea aphids, *A. pisum*, might be associated with variation in energetic reserves [49]. In the case of ACP, it was initially hypothesized that abdominal color variation might be associated with the quantity or quality of the acquired food resources [47]. Recently, Ramsey et al. [50] found that the ACP genome contains three predicted hemocyanin genes that are differently expressed in the different color morphs. Hemocyanins are oxygen transport proteins found exclusively in mollusca and arthropods, the blue color being imparted by the molecules of copper. Hemocyanin 1 expression was three-fold higher in the blue morph ACP than the grey morphs, and was nearly undetectable in yellow morphs [50]. The extended flight ability of the blue-green morph might be due to the higher level of hemocyanins, resulting in additional oxygen-carrying capacity, during flight. While orange males exhibit higher fecundity, blue males appear more capable in performing long-range dispersal [18]. Color of male abdomens has been linked to the amount of fat body in the abdomen [45]. Recently, Ibanez et al. [51] demonstrated that higher reproductive output of blue/green than gray/brown female *D. citri* is specifically associated with expression of the genes *VgA1-like and Kr-h1*, which occurs during oocyte development. Furthermore, *VgA1-like* exhibited female-specific expression [51].

#### 3.3.8. Effects of Candidatus Liberibacter Asiaticus (CLas) on dispersal

##### Indirect Effects of *C*Las on Movement

ACP females infected with *C*Las are more attractive to conspecific males than their uninfected counterparts [52]. Additionally, *C*Las-infected trees are more attractive to both sexes of ACP, as compared to uninfected trees [38]. However, this vector preference for infected plants decreases over time as disease progresses, which coincides with reduced release of MeSA [52]. Nonetheless, plant pathogen manipulation of host plant volatiles to attract their vectors might increase the spread of *C*Las throughout citrus orchards [53]. The possible impact of pathogen infection on mating ACP requires more investigation.

##### Direct Effects of CLas Pathogen on Movement

CLas acquisition changes inclination for dispersal, flight capacity, and sexual attraction of ACP [54]. These effects increase movement of the infected ACP to promote spread of the pathogen. There are several examples of coevolved mechanisms between pathogens, hosts, and vectors, in which pathogens manipulate hosts in a manner predicted to favor the transmission and spread of pathogens [55,56,57].

Spread of phytopathogen-mediated diseases is often associated with the frequency of vector dispersal [58,59,60,61,62]. Increased frequency of short-distance flights by ACP is likely to increase inoculation frequency of the same citrus host at different locations, whereas increased frequency of long-distance flights might increase the spread of pathogen to new and distant areas. *C*Las infection increases the probability of both short and long-distance flights by ACP [54], but the physiological limits of the vectors flight ability likely limit the impact of these behavioral changes on pathogen spread [54].

### 3.4. Abiotic Drivers of Dispersal

#### 3.4.1. Temperature and Humidity

Dispersal of ACP increases linearly with temperature (within the range of ca. 18–33 °C) and independent of the relative humidity level [63]. However, wing shape and temperature are strongly correlated with larger and broader wings observed at colder temperatures, under laboratory rearing [48]. Changes in relative humidity (RH) or the interaction between RH and temperature do not affect ACP flight [64]. The threshold for ACP flight initiation is approximately 16.5 °C [63]. Females are stronger fliers than males and gray-brown morphs generally disperse more than blue-green morphs [63].

#### 3.4.2. Barometric Pressure

There is a significant positive relationship between frequency of ACP dispersal and increasing barometric pressure from 1,009 to 1,022 mbar [63]. Least dispersal occurs when the pressure drops at −5.42 mbar and most dispersal occurs with pressure increases at 4.57 mbar/h [63]. Under steady pressure (between −0.3 mbar to 0.3 mbar/h variation), ACP disperses less than during pressure increases. ACPs are less active during decreasing than increasing barometric pressure [63].

These results would indicate that ACP can forecast weather and adapt behavior to maximize survival. Rapid drops in the barometric pressure usually presage inclement weather conditions and ACP responds by inhibiting flight initiation. Interestingly, Zagvazdina et al. [64] found contrasting results with different behaviors, in response to a pressure decrease 24 h prior to testing, i.e., increased phototaxis but reduced mate-seeking behavior. Thus, the response to pressure drops (increasing or decreasing activity) might vary, depending on the specific behavioral response that is measured.

#### 3.4.3. Wind and Elevation

Abundance of ACP has been found to decrease with increasing elevation [65,66]; however, more recent observations in Puerto Rico appear to contradict these claims (P. Stansly upublished data). Wind likely facilitates movement of ACP within lower jet streams between islands [20]. Although, mark–recapture investigations thus far have failed to demonstrate that ACP movement is aided by wind within an agricultural landscape [14], detailed laboratory and subsequent field trapping investigations have indicated that ACP fly along with wind; flight is greatest at wind speeds of 0.3 m/s and decreases steadily above this optimum (Martini et al. unpublished). Other abiotic factors, such as habitat heterogeneity or drought could also affect ACP dispersal [25,67] and deserves further research.

#### 3.4.4. Anthropogenic Movement and the Urban Landscape

Recent analyses of ACP geographic and temporal population dynamics in Southern California have demonstrated non-random and statistically significant associations between population “hotspots” and urbanization [68]. Urban areas in Southern California were specifically associated with population hotspots, suggesting that these areas might have served as a source of population introductions. The mechanisms suggested by Bayles et al. [68] included more frequent transport of plant material, including possible unregulated/illegal plants, coupled with abundance of hosts (i.e., residential citrus) associated with urban areas.

#### 3.4.5. Landscape Architecture

ACP population densities are much higher in solidly planted orchards comprised of seedling trees than in orchards where seedlings are replanted among otherwise surrounding mature trees [25]. This indicates that removal of individual infected trees and replacement with clean stock should promote lower ACP populations than replacement of entire orchard blocks. It is possible that differences in microclimate might affect ACP reproduction on young reset trees compared with those replanted in solid sets [25]. For example, reset trees benefit psyllids by providing moderate climate characteristics, such as shade and wind protection. In addition, seedling trees grown in solid sets produce more abundant young leaf resources for ACP reproduction than interspersed resets [69]. It has been demonstrated that ACP immigration into orchards is positively correlated with abundance of new leaf flush [14]. Favorable microclimate and habitat complexity are likely to both influence ACP population densities within orchards.

#### 3.4.6. Windbreaks

Windbreaks planted around orchard edges reduce ACP densities along borders and within citrus orchards. Windbreaks do not appear to affect populations of ACP’s natural enemies within orchards [25]. It is possible that windbreaks affect microclimate to reduce ACP populations. For example, windbreaks might reduce leeward wind elevation and night-time temperature while increasing the daytime temperatures near the windbreak on the leeside, compared to temperatures further away from the windbreak [70,71].

## 4. Conclusions and Opportunities for Future Improvements of HLB Management

### 4.1. Management and Quarantine

Introduction of ACP and HLB has challenged the previous norm of managing exotic pest introductions in U.S. citrus production, through rapid spread, difficulty in detection, and lack of preventive or therapeutic treatments. Citrus industries throughout the US responded to the appearance of ACP and HLB, by coordinating committed funding within and between citrus states, to initiate new research. They also enlisted help in coordination, response, funding from USDA–ARS and USDA–APHIS. Emergent from this request for federal help was the evolution of APHIS Citrus Health Management Program (CHRP) in July 2006, addressing all citrus states. There was also large-scale redirection of funding from marketing and other self-funded programs to fuel and accelerate the needed research efforts. Florida has spearheaded research efforts to understand the HLB problem and initially sought assistance (CA, TX involved) from the National Research Council of NAS and the USDA–ARS National Program Leaders to, (1) assist in prioritizing within-year research projects for immediate funding, in 2008, (2) develop short, medium, and long-term research priorities to address HLB/ACP, and (3) develop a management plan for industries to organize, manage, and communicate research projects and results. The strategic plan was published by NAS in 2010 (www.nap.edu/catalog/12880.html, ISBN: 0-309-15208-9). The first research priority was to “improve insecticide-based management of Asian citrus psyllid (ACP)”. The first organizational recommendation was to “create ‘Citrus Health Management Areas’ in Florida”. Area-wide management was predicted to reduce immigration of the infected ACP into commercial citrus, with the understanding that ACP can invade from distances of at least 2 km, without wind assistance. Implementing the practical use of any single or multiple-integrated technologies requires large-scale cooperation of many growers, on a large scale, to overcome the natural and man-assisted dispersal capabilities of this vector. Quarantine protocols must also account for the possibility of hundreds of kilometers of movement by the vector, even when these long-range movements occur by man- or wind-assisted movement events.

### 4.2. Abandoned Citrus and Management

Abandoned citrus serves as a reservoir of ACP and *C*Las and should be considered when implementing an area-wide management plan. The area of abandoned citrus in Florida has increased since 2008 because of socioeconomic pressures, freezes, and the citrus produce loss caused by diseases [72]. Abandoned orchards are not managed with agrochemicals and populations of ACP go unchecked. Abandoned citrus is a reservoir for both pathogen and vectors [11]. The direct impacts of abandoned orchards on commercial citrus production is difficult to quantify. This issue requires quantification of both, in areas where HLB is encroaching on commercial production, as well as in residential areas where citrus is grown by homeowners. While intensity and coordination of control measures for ACP might reduce impacts of HLB, sources of inoculum from abandoned orchards could undermine these efforts [73].

In response, recommendations have been proposed to remove and destroy abandoned citrus [74]. This has led to reductions of abandoned orchards in Florida [74,75]. Management of an adjacent abandoned citrus acreage is often recommended to growers who own citrus adjacent to unprotected citrus. Geographical barriers appear not to discourage ACP dispersal; for example, psyllids have been captured in a dense forest 2.3 km away from known host plants [12].

Recently, the “USDA’s Tree Assistance Program” was initiated in response to the record low citrus production in Florida in 2014 [76]. The purpose of this program is to subsidize planting of new citrus trees to replace those lost to HLB. This newly planted area will comprise solidly planted seedlings, presenting significant challenges for vector management. Cultural control tools such as windbreaks and reflective mulches are recommended to help contain ACP populations and to lessen reliance on intense insecticide input [77].

Implementation and conservation of windbreaks is one component of current integrated pest management for ACP and associated HLB, [25]. Despite costs associated with windbreaks, such as competition for water and loss of nutrient-rich land, windbreak protection against other diseases such as citrus canker, in addition to psyllid reduction [70] likely outweigh the disadvantages. In addition to natural windbreaks to reduce ACP immigration, growing citrus confined within artificial structures that completely exclude the vector is gaining momentum in commercial citrus production.
